# Pathways to Tailor Photocatalytic Performance of TiO_2_ Thin Films Deposited by Reactive Magnetron Sputtering

**DOI:** 10.3390/ma12172840

**Published:** 2019-09-03

**Authors:** Alexander Vahl, Salih Veziroglu, Bodo Henkel, Thomas Strunskus, Oleksandr Polonskyi, Oral Cenk Aktas, Franz Faupel

**Affiliations:** Institute for Materials Science—Chair for Multicomponent Materials, Faculty of Engineering, Kiel University, Kaiserstraße 2, D-24143 Kiel, Germany (A.V.) (S.V.) (B.H.) (T.S.) (O.P.)

**Keywords:** titanium dioxide, photocatalysis, thin films, nanoparticles, sputtering

## Abstract

TiO_2_ thin films are used extensively for a broad range of applications including environmental remediation, self-cleaning technologies (windows, building exteriors, and textiles), water splitting, antibacterial, and biomedical surfaces. While a broad range of methods such as wet-chemical synthesis techniques, chemical vapor deposition (CVD), and physical vapor deposition (PVD) have been developed for preparation of TiO_2_ thin films, PVD techniques allow a good control of the homogeneity and thickness as well as provide a good film adhesion. On the other hand, the choice of the PVD technique enormously influences the photocatalytic performance of the TiO_2_ layer to be deposited. Three important parameters play an important role on the photocatalytic performance of TiO_2_ thin films: first, the different pathways in crystallization (nucleation and growth); second, anatase/rutile formation; and third, surface area at the interface to the reactants. This study aims to provide a review regarding some strategies developed by our research group in recent years to improve the photocatalytic performance of TiO_2_ thin films. An innovative approach, which uses thermally induced nanocrack networks as an effective tool to enhance the photocatalytic performance of sputter deposited TiO_2_ thin films, is presented. Plasmonic and non-plasmonic enhancement of photocatalytic performance by decorating TiO_2_ thin films with metallic nanostructures are also briefly discussed by case studies. In addition to remediation applications, a new approach, which utilizes highly active photocatalytic TiO_2_ thin film for micro- and nanostructuring, is also presented.

## 1. Introduction

Among other semiconductors, TiO_2_ is the most extensively used photocatalyst for environmental remediation (e.g., water cleaning and air purification) and energy harvesting (e.g., water splitting for hydrogen generation) applications due to its low cost, chemically inertness, non-toxicity, high photocatalytic activity, and recyclability [1]. On the other hand, the high band gap of TiO_2_ (3.0 eV for rutile and 3.2 eV for anatase), makes it photoactive only under ultra-violet (UV) radiation. TiO_2_ exhibits different polymorphs (anatase, brookite, and rutile) among which anatase is known as the most efficient for photocatalytic applications [2]. Moreover, the thermodynamic and structure-based analysis showed that anatase is the most stable TiO_2_ phase at nanoscale due to its relatively lower surface energy [3].

Anatase nanoparticles (NPs) have been almost accepted as the “golden standard” photocatalyst in terms of their high pollutant (organic dyes, other pollutants) degradation capacity in water [4]. On the other hand, separating such extremely tiny particles from an aqueous medium is not an easy task which needs additional technology which leads to further cost. Instead, the use of TiO_2_ thin films is more suitable for water treatment, as well as other environmental remediation applications such as air and odor cleaning [5].

TiO_2_ thin films can be prepared by different approaches such as physical vapor deposition (PVD) (including thermal evaporation, reactive sputtering, ion or electron beam evaporation), chemical vapor deposition (CVD) techniques, and as well as by wet chemical deposition methods (dip-coating, spin-coating, spray coating, etc.) [6]. Due to their low cost and ease of up-scaling, wet chemical methods are preferred especially for outdoor applications (self-cleaning textiles or exterior walls of buildings) of TiO_2_ based coatings. On the other hand, such methods mostly need to be followed by secondary processes such as drying and annealing for attaining stable and especially crystalline TiO_2_ layers, which is essential to achieve a high photocatalytic performance. In general, vapor phase methods provide various advantages such as well controlled homogeneity and thickness over a large area and good adhesion [7]. Basically, CVD processes run at much higher temperatures (400–900 °C) in comparison to PVD processes [8]. This excessive heating limits the use of CVD for some technical applications where substrate material cannot tolerate high temperatures. Moreover, it is known that some titanium-based precursors and their by-products are highly corrosive, which lead to various material handling and storage problems [9]. In recent years for the preparation of TiO_2_ thin films, PVD methods gained enormous interest since they are not limited to the deposition only at thermodynamically equilibrium and they run at much lower costs in comparison to CVD processes [10].

Various PVD methods including electron beam evaporation, bias assisted cathodic arc deposition, oxidation process, magnetron sputtering, pulsed laser deposition (PLD), and thermal evaporation have been explored for deposition of TiO_2_ thin films [11]. Commonly, films of high density and homogeneity are obtained by all PVD methods in which the growing film is bombarded with energetic particles, as is the case for sputtering [12]. DC magnetron sputtering of TiO_2_ films is of special interest, first of all, because it is an industrial process applicable to large-area deposition. The method allows high-quality TiO_2_ films to be achieved, even at low substrate temperatures. For instance, roll-to-roll system sputtering systems allows coating of TiO_2_ and similar oxides on several tens-to-hundreds of meters long flexible organic and inorganic films. On the other hand, TiO_2_ thin films prepared by different PVD techniques exhibit diverse photocatalytic efficiencies. Such a difference is clearly seen, especially between evaporated and sputter deposited TiO_2_ thin films [10]. This difference can be attributed to diverse film characteristics including surface area, morphology, defect density, and crystallization pathway.

In comparison to colloidal TiO_2_ NPs, TiO_2_ thin films have a very limited surface area which impedes efficient photocatalytic decomposition of organic pollutants [13]. Therefore, various strategies have been proposed to enhance the surface area of TiO_2_ thin films to achieve high photocatalytic performance. Novel sputtering and evaporation methods have been developed to achieve columnar or sculptured thin films with high porosity (determined by column diameter and spacing) [14]. Suzuki et al. demonstrated an enhanced surface reaction efficiency of obliquely deposited TiO_2_ thin films with differently shaped columns including zigzag, cylinder, and helix morphologies [15]. As an alternative to 2D films, Goossens et al. reported a fractal (‘forest-like’) 3D TiO_2_ thin film to achieve higher photocatalytic efficiency [16]. Totally different to the oblique deposition approach, we combined gas aggregation source (GAS) and sputtering methods to achieve a TiO_2_ thin film composed of a TiO_2_ NPs top layer (enhancing the active surface area) and a columnar TiO_2_ film underneath. While the top NPs layer enhances photocatalytic efficiency, the bottom layer provides high stability. Recently, we demonstrated another effective process, which basically allows controlled nano-crack network formation within sputter deposited TiO_2_ thin films, to achieve a high surface area [17]. The choice of the deposition method type plays not only a major role in the thin film morphology, but it also defines the crystallization pathway [10].

Elemental doping is one of the most common approaches to enhance the catalytic/photocatalytic properties of TiO_2_. Various dopant materials have been used to modify the band-gap of TiO_2_ for extending its activity to longer wavelengths. These dopants can be metals (noble and transition metals), as well as non-metals, including elements such as N, B, S, and C [18,19]. Noble metals such as Ag, Au, Pd, and Pt exhibit superior absorption properties owing to the surface plasmon resonance (SPR), but their high cost limits their use, especially in large scale applications [20]. In this connection, transition metals provide a more cost-effective approach [21]. On the other hand, these metals suffer from instability due to their fast leaching characteristics. Therefore, non-metals are heavily preferred in doping of TiO_2_. In this review, doping was left out of the focus.

Decorating TiO_2_ thin films with metallic nanostructures such as Au and Ag NPs is another effective approach to enhance photocatalytic efficiency through the localized surface plasmon resonance (LSPR) effect [22]. TiO_2_ thin film decorated with Au and Ag exhibited red-shifted and intensified plasmon resonance, which improved the overall photocatalytic performance. On the other hand, metallic NPs can also enhance the photocatalytic performance of TiO_2_ thin films through a non-plasmonic mechanism [23]. Basically, metallic NPs deposited on TiO_2_ thin film can act as electron-sink centers, which improve the charge separation of photoexcited TiO_2_.

In this paper, a particular emphasis is placed on TiO_2_ thin films prepared by PVD methods and some specific strategies (basically developed at our laboratory in recent years) which aim enhancing the photocatalytic performance of such thin films. First, we focused on the morphology of the thin film and its influence on the photocatalytic performance. We compared morphologies of TiO_2_ thin films deposited by different PVD methods and also present innovative approaches for increasing the surface area of TiO_2_ thin films. Then, we concentrated on the improvement of the photocatalysis by decorating TiO_2_ thin films metallic NPs. While discussing both plasmonic and non-plasmonic contributions of such NPs, we also demonstrated case studies from visible (Vis) and UV plasmonic enhanced photocatalysis. Finally, we presented a new approach which uses highly active TiO_2_ thin film as a functional tool for micro- and nanostructuring of surfaces.

## 2. Sputter Deposited and Evaporated TiO_2_ Thin Films

Here we mainly discuss the deposition of the TiO_2_ thin films by electron beam evaporation and pulsed unipolar DC magnetron sputtering from a metallic target in a reactive O_2_/Ar atmosphere to understand their differences in terms of the microstructure and resulting properties affecting photocatalysis. We would like to mention that we focused on comparing only mentioned methods rather than giving a general review of PVD methods. Technical details about the deposition of TiO_2_ thin films by electron beam evaporation can be found in our previous work [10] and details about the deposition by reactive sputtering from a metallic Ti target is described in our earlier publications [17,24]. These studies allowed us to derive the following conclusion; basically, three parameters play an important role in the photocatalytic performance of TiO_2_ thin films: first, the different pathways in crystallization (nucleation and growth); second, anatase/rutile formation; and third, surface area at the interface to the reactants. Basically, the shape of TiO_2_ structures, which is a critical parameter for determining the photocatalytic activity, is governed by nucleation and growth pathways. Recent research efforts have shown the importance of tailoring the crystal shape of TiO_2_ and as well as other photocatalysts to achieve more reactive facets [25,26]. For instance, the ratio between {101} and {001} surfaces strongly determine the photocatalytic performance of TiO_2_ [27].

**Figure 1:**
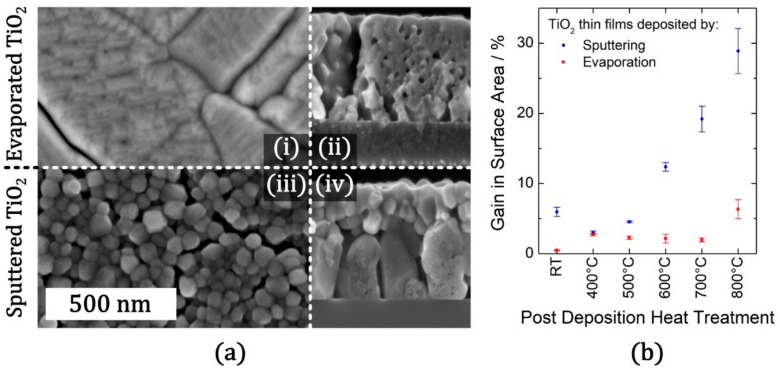
(**a**) SEM micrographs (having same scale bar of 500 nm) in top view ((i) + (iii)) and cross section ((ii) + (iv)) configuration of TiO_2_ thin films post heat treatment (800 °C 1 h): (i) + (ii) evaporated TiO_2_; (iii) + (iv) sputtered TiO_2_. (**b**) Gain in surface area for sputter deposited or evaporated TiO_2_ thin films compared to an ideally flat thin film, calculated from atomic force microscopy (AFM) measurements (thin films have no open porosity). The error bars correspond to three standard deviations (3σ). Adapted with permission from [10]. Copyright (2016) Elsevier.

When we compare sputter deposited and evaporated TiO_2_ thin films in terms of their structural characteristics, both approaches lead to amorphous layers with columnar structures [10]. On the other hand, the columnar morphology is more predominant in the case of sputter deposited TiO_2_ films. Actually, in our detailed study, we reported clear differences between the morphologies of evaporated and sputter deposited TiO_2_ films. During evaporation at room temperature, incoming vapor flux has low energy (thermal energy α k_B_T), therefore the growing layer is far away from the melting temperature (T_m_). So far, from the point of thermodynamic equilibrium, the nucleation barrier is low in case of evaporated films, which leads to the classical nucleation and the grain growth throughout the bulk [28]. At the early stages of the growth, we observed a high grain density with uniform distribution of equal-sized grains. This was followed by the out-of-plane growth of such grains in accordance with the growth model suggested by Van der Drift et al. [28]. Indeed, the columnar texture results from the difference in growth rates between different crystal faces of the grains on the film surface. Grains oriented with their faster-growing directions perpendicular to the surface stay intact while slower growing grains are terminated as they intersect the column walls of taller grains. This also triggers the constitution of voids into closed porosity thought such columnar structures at 800 °C at 1 h time at temperature (TAT), as shown in Figure 1.

In contrast to evaporated TiO_2_ layers, sputtered layers quickly heat up to about several hundred °C during the deposition by the transfer of kinetic energy of incoming atoms and ions [10]. Due to being significantly closer to a thermodynamic equilibrium (compared to evaporated films), low driving force impedes the nuclei formation (homogenous nucleation) in sputter deposited films. Therefore, there is a need for favorable nucleation sites facilitating the film growth. Basically, the nuclei forms at the interface, then grows out-of-plane as a buried layer beneath a distinct top layer as shown in Figure 1. We reported that the latter stays nearly amorphous up to tempering temperature around 500 °C. On the other hand, at higher temperatures, the nucleation barrier can be tackled throughout the whole bulk (regardless of being any favorable nucleation site), which lead to dense nucleation, uncontrolled grain growth, and Ostwald ripening at the top layer.

Since there is a clear difference in terms of nucleation and growth mechanisms between sputter deposition and evaporation, one should also consider a difference in their crystallization. The sputtered layers exhibit heterogeneous crystallization, while the evaporated layers undergo homogeneous crystallization. Here, crystallization type plays an important role in promoting anatase formation at the thin film-reactant interface, which is crucial for achieving a high photocatalytic efficiency [29,30]. Due to the homogeneous crystallization, anatase is the predominant phase throughout evaporated thin films and this leads to high photocatalytic performance already at lower heat-treatment temperatures [31]. In contrast, in sputtered layers an effective anatase interface (in contact with reactants) is missing; only buried anatase grains may provide charge carriers upon radiation, but their random walk to the surface is hindered by the defect-rich top layer. Therefore, up to 500 °C, the sputtered thin films have half the efficiency and only upon nucleation in the top layer starting above 500 °C they surpass evaporated films by far.

Despite a small synergetic ratio regime of anatase to rutile [32], which is also still controversially debated in the literature [29], rutile is known to reduce the photocatalytic efficiency dramatically [30,33]. Our Raman spectroscopy analysis of the sputtered thin films revealed that rutile phase starts appearing at 600 °C 1 h TAT and is quite pronounced at 800 °C 1 h TAT [17]. Formation of some initial rutile nuclei is promoted by the higher deposition temperature during the sputtering. However, during the grain growth anatase is more favorable at first, because anatase has lower surface energy than rutile (at first the driving force is higher to form high volume to surface ratio of anatase). At higher temperatures, growth barriers are low enough for causing that the existing rutile nuclei become obvious. In contrast, we observed that evaporated thin films do not exhibit a rutile phase in Raman spectra all up to 800 °C 1 h TAT. Initial rutile nuclei are negligible during evaporating at room temperature. During the heat-treatment of evaporated thin films, there are three factors promoting anatase in favor over rutile. Rutile has a higher nucleation barrier, so homogeneous nucleation of anatase is favored. Even for the statistical few existing rutile nuclei, the factor of lower anatase surface energy affects the growth as stated already for sputtered thin films. Third, the void formation during the grain growth makes the reconstruction of the existing anatase grains into ones with lower surface area more favorable before transforming into rutile. While the photocatalytic efficiency of sputter deposited thin films decreases intensely with rutile formation, evaporated thin films exhibited a further increase in photocatalytic efficiency due to the absence of rutile up to 800 °C 1 h TAT. At 600 °C 1 h TAT (just before their decline), sputter deposited thin films showed significantly higher photocatalytic efficiency than evaporated ones (even exceeding the maximum efficiency observed for evaporated thin films). For sputter deposited thin films, 600 °C seems to be high enough for crystallization of the top layer, but it is still low for promoting rutile formation. This may explain such a high photocatalytic efficiency (43% higher than evaporated thin films). However, we believe the huge difference in the photocatalytic efficiencies arises from the large effective surface area (at the catalytic interface) of sputter deposited thin films. AFM analysis (gain in surface area (%)) illustrated in Figure 1 indicates that evaporated thin films stay comparably smooth over all heat-treatment temperatures, while the sputtered thin films have a significant increase in surface area at higher temperatures.

Measuring with an industry standard UV-LED, good for photocatalytic reactors which were one part of our studies, irradiance is not the limiting factor, in contrast to e.g., the case of ambient indoor light applications. Availability of adsorbed reactants and transport of products are limiting factors here, causing the first-order reaction for photocatalysis. Therefore, a larger surface area helps to achieve a high photocatalytic efficiency, since the capacity for reactant adsorption is proportional to the specific surface area [30]. If rutile formation could be suppressed in these sputtered thin films up to higher temperatures, it is likely that their efficiency would increase further up to 800 °C 1 h TAT, which is an interesting lead for further experiments.

In brief, their significantly higher photocatalytic efficiency made us focus on sputtered thin films. We presented some possible approaches to further increase the surface area at the catalytic interface and as well as other pathways to enhance the photocatalytic activity of sputter deposited TiO_2_ thin films in the next section.

## 3. Pathways for Enhancing Photocatalytic Performance of Sputter Deposited TiO_2_ Thin Films

Basically, photocatalysis describes the process of generating electron-hole pairs within a semiconducting material upon irradiation by photons and the subsequent facilitation of chemical reactions mediated through photo-generated electrons or holes at the surface of the material [34]. From this idealized picture, two main approaches to enhance the photocatalytic performance of a given material become obvious: first, the yield of photo-generated electrons and holes arriving at the semiconductor surface can be tailored by an increase in the time constant for recombination, e.g., by reducing the amount of defects in the crystalline material, which would act as recombination sites [35]. Second, the transition from a planar thin film to two-dimensional (2D) or three-dimensional (3D) nanostructures leads to a significant increase in the effective surface area (which the photocatalytic reaction can take place) and it simultaneously decreases the characteristic length scale towards the mean free path of photo-generated electrons and holes (increase in the yield of charge carriers that successfully arrive at the semiconductor surface) [17].

Particle-based photocatalysts successfully follow the latter approach and reach very high photocatalytic performances when they are applied as colloids. Although conventional thin film photocatalysts generally tend to be outperformed by their colloidal NPs counterparts, in terms of mechanical stability and long-term usability, they are promising for various functional applications. On this background, the investigation of TiO_2_ thin films and nanocomposites with tailored morphologies is essential to merge the mechanical stability of thin films and the high surface area of nanostructures. Accordingly, in this section, different concepts for the enhancement of the photocatalytic performance of sputter deposited TiO_2_ thin films will be explored.

### 3.1. Surface Decoration of Sputter Deposited TiO_2_ Thin Films with NPs

**Figure 2:**
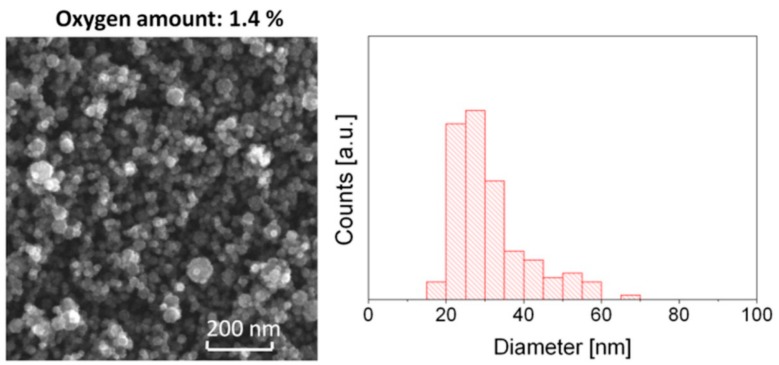
SEM images and corresponding size histograms of TiO_2_ nanoparticles (NPs) deposited using Ar/O_2_ mixture with 1.4% of O_2_. Adapted with permission from [37]. Copyright (2012) Elsevier.

In comparison to TiO_2_ colloidal particles, TiO_2_ thin films have a very low surface area. One can decorate the surface of sputter deposited TiO_2_ thin films with TiO_2_ NPs to increase the reactive surface area. Suresh et al. reported such an approach which covers the deposition of TiO_2_ NPs by sol-gel method [36]. One of the challenges in using TiO_2_ colloidal NPs is achieving a strong adhesion between these particles and the TiO_2_ thin film. As an alternative to the wet chemical synthesis of TiO_2_ NPs, solvent-free methods have been also reported, for instance, Biederman et al. reported a novel approach for the synthesis of TiO_2_ NPs using a GAS, as shown in Figure 2 [37]. Similarly, Ghori et al. and Polonskyi et al. showed the effectiveness of GAS in preparation of different NPs [31,38]. In all these approaches the authors claimed a strong adhesion between deposited NPs and the substrate.

**Figure 3:**
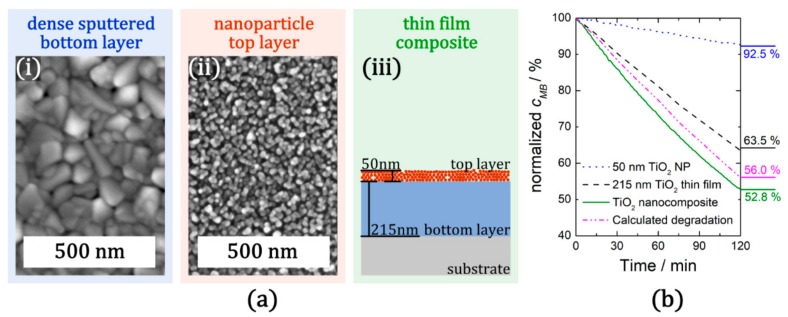
TiO_2_ NPs/TiO_2_ nanocomposites for enhanced photocatalytic performance: (**a**) SEM top view micrographs of the composite layers (i: bottom layer; ii: top layer) and schematic cross section of the thin film composite (iii); (**b**) photocatalytic performance of the nanocomposite (green line) as well as its constituent layers (blue line: NPs top layer; black line: dense bottom layer). Compared to the calculated degradation of the nanocomposite (magenta line, the addition of degradation of the individual components), the manufactured nanocomposite exhibits an increased photocatalytic performance, which implies a synergistic effect of surface decoration of dense TiO_2_ thin films by TiO_2_ NPs. (details are given in Supplementary Materials).

The mechanical stability of a TiO_2_ thin film and the high surface area of TiO_2_ NPs can be combined within a nanocomposite layer that features both individual components. For this purpose, we first prepared a TiO_2_ thin film by reactive sputtering and following a heat-treatment step (Figure 3a (i)) we deposited a continuous layer of TiO_2_ NPs (Figure 3a (ii)) using GAS method (details are given in Supplementary Materials). Here, we used a Haberland type GAS which allows deposition of TiO_2_ NPs with high purity and good process control. Combining these two deposition methods, TiO_2_ NPs/TiO_2_ nanocomposites can be fabricated by consecutive PVD steps, as exemplarily depicted in Figure 3a (iii).

The morphology and the schematic representation of the nanocomposite composed of a top layer containing a NPs layer (with a nominal thickness of 50 nm) and a base TiO_2_ thin film with a nominal thickness of 215 nm are presented in Figure 3. TiO_2_ NPs form a porous layer and have an individual mean diameter of almost 16 nm (details are given in Supplementary Materials). The photocatalytic performance (degradation of methylene blue, MB) of the nanocomposite and its individual components under UV irradiation is shown in Figure 3b. The direct comparison reveals that the composite (green line) performs better than the TiO_2_ thin film base layer (black line) as well as NPs layer (blue line) or the calculated sum of the two components (magenta line). Accordingly, there has to be a synergistic effect upon combining the TiO_2_ thin film and the TiO_2_ NPs. This effect can be explained as follows: the dense bottom layer generates electron-hole pairs with high efficiency (and low recombination) and can transfer these to the top NPs layer, which in turn offers a high surface area and consequently sufficient reaction sites for the photocatalytic degradation of MB. Its mechanical stability makes the nanocomposite layer a potential coating material for various applications. The nanocomposite comprising of a sputtered TiO_2_ thin film decorated with TiO_2_ NPs endures multiple cleaning cycles in an ultrasonic bath. In contrast, TiO_2_ NPs directly deposited onto quartz glass substrates exhibited an insufficient adhesion which leads to the detachment of NPs already after the first cleaning cycle. This is reminiscent of the same situation mostly observed in the case of spin-coated or dip-coated colloidal TiO_2_ NPs on similar substrates [39].

### 3.2. Thermally Induced Nanocrack Network Formation

**Figure 4:**
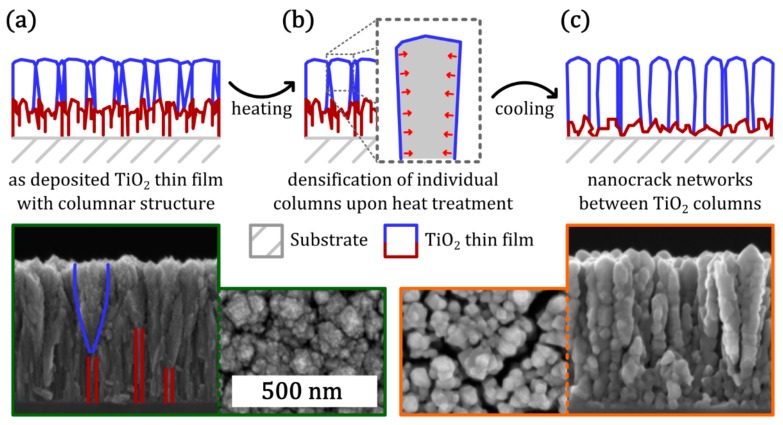
Overview on the process of nanocrack network formation in TiO_2_ thin films deposited by sputter deposition: (**a**) schematic cross-section of the as-deposited TiO_2_ thin film; (**b**) morphologic changes upon heat treatment; (**c**) schematic cross-section of the TiO_2_ thin film with characteristic nanocrack networks formed after heat treatment. (SEM micrographs (having same scale bar of 500 nm) given at the bottom show in cross-sectional as well as top view configurations for the as-grown (green border) and the heat treated (orange border) TiO_2_ thin films). Adapted with permission from [17]. Copyright (2018) IOP.

Instead of increasing the effective surface area by applying a layer of TiO_2_ NPs, the base TiO_2_ layer itself can be nanostructured in order to increase the photocatalytic performance. In a recent approach, we reported self-organization of a network of nanoscopic cracks within a TiO_2_ thin film that was deposited by reactive sputtering and subjected to a post-deposition heat treatment [17]. The mechanism of nanocrack formation (schematically depicted in Figure 4) in TiO_2_ thin films deposited by pulsed D.C. reactive sputtering can be explained as follows:

- In the as-deposited state, the TiO_2_ thin film contains two distinct morphological features: in the vicinity of the interface with the substrate, there is a highly defective layer, which is characterized by irregular and narrow columns (indicated by red lines in Figure 4a). At a certain distance from the interface to the substrate, the dominating features turn into a columnar, partially crystalline anatase layer with broader columnar structures (indicated by blue lines).
- Upon heating, the TiO_2_ thin film difference is subjected to compressive stress due to the mismatch in thermal expansion coefficients between the substrate and the thin film. The TiO_2_ columns (which are already present in the as-deposited thin film) crystallize and get denser, which leads to reduction of the total compressive stress on the thin film. The high saturation by oxygen prevents the individual columns from coalescence.
- Subsequent to the heating step, the TiO_2_ thin film sample is cooled to room temperature and the stress in the thin film is relaxed. Due to the densification of the individual columns, the thin film is now subjected to tensile stress, which results in crack formation. These cracks preferentially are formed along the flanks of individual TiO_2_ columns, which results in the presence of nanocrack networks in the TiO_2_ thin films post heat treatment (as depicted in the SEM micrographs in Figure 4c).

As described the formation of the nanocrack network in the model, the structural features and the morphology of the as-deposited thin film are crucial to the formation of nanocrack networks. The occurrence of these structural features, namely dense and defected columns in the vicinity of the interface to Si substrate and broad and partially crystalline columns at a further distance from the interface, can be attributed to the deposition process of reactive sputtering itself. For deposition of TiO_2_ thin films (with nanocrack networks) by reactive sputtering, typically a reactive gas atmosphere with an oxygen flow of 10 SCCM and an argon flow of 250 SCCM is applied. Accordingly, there is a high oxygen amount in the reactive atmosphere and metallic titanium exhibits a strong tendency to oxidize. As the deposition is initialized, the substrate surface offers a high number of available nucleation sites. The high availability of reactive oxygen species leads to the growth of a stoichiometric TiO_2_ thin film as the deposited Ti species are readily saturated by oxygen. These saturated bonds as well as the low surface diffusion prevents significant coalescence of columns or seeds. Therefore, in the initial stage of deposition, the thin film is characterized by a high number of columns with high defect density. Due to the limited thermal conductivity of the deposited TiO_2_ thin film, the temperature at the deposition front increases gradually with ongoing deposition. As a result, anatase crystallites start to form and the dense, defected columns turn into conical shaped grains.

**Figure 5:**
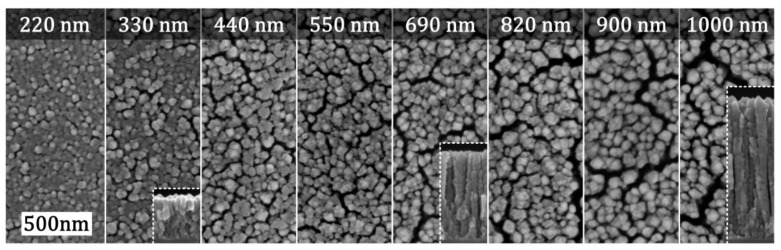
SEM micrographs of TiO_2_ thin films with layer thicknesses ranging from 220 to 1000 nm. Considerable nanocrack networks are observed for layer thicknesses of 440 nm and above. The insets show cross-sectional micrographs. Adapted with permission from [17]. Copyright (2018) IOP.

In the exemplary TiO_2_ thin film presented in Figure 5, this transition occurs roughly at a distance of 200 nm from the substrate interface. Accordingly, the extent of nanocrack network formation is expected to depend on the thickness of the deposited TiO_2_ thin films, which is underlined by the comparison of SEM micrographs in Figure 5. While for low layer thicknesses, there are only individual cracks, for higher layer thicknesses above 440 nm a continuous network of cracks is formed. The extent of nanocrack formation is even higher for increasing layer thicknesses, as such the thin films with a thickness of 1000 nm show the most pronounced nanocrack network with the highest crack widths.

Alongside the evolution of nanocrack networks in TiO_2_, the effective surface area is also significantly enhanced and a beneficial effect on the photocatalytic performance is reported [17]. However, a direct correlation between the extent of nanocrack formation and the enhancement in photocatalytic performance is not trivial. With increasing layer thickness, the formation of cracks is increased as well as the amount of material for the generation of electron-hole pairs. In general, the film thickness influences three competing factors taking place in photocatalytic reactions: (i) the area available for chemical reactions, (ii) the transport of the solute and charge carriers, and (iii) the light distribution [40]. While the thickness increases, the total area available for the chemical reaction also increases. In contrast, the electron-hole transport within the film becomes difficult with increasing film thickness. According to Beer–Lambert’s law, the light attenuation becomes more pronounced as the film thickness increases [40]. Therefore, it is important to achieve an optimal film thickness.

In order to reveal the effect of nanocrack networks on the photocatalytic performance, a fitting model is under consideration, which includes the geometrical effect of higher surface area as well as the increasing amount of material. On the basis of this model, indeed, a beneficial effect of nanocrack networks could be observed [17]. However, as given above, understanding the direct effect of the film thickness (beside crack formation mechanism) on the photocatalytic performance is not trivial.

**Figure 6:**
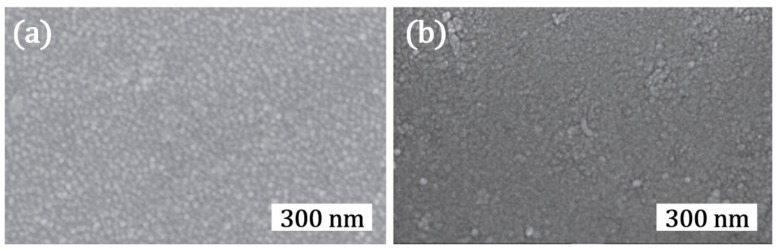
SEM top view micrographs of TiO_2_ thin films deposited on glass (**a**) as deposited and (**b**) annealed at 200 °C. Adapted with permission from [41]. Copyright (2016) IOP.

We have shown that this controlled crack formation can be effectively used to prepare highly photocatalytic TiO_2_ thin films [13]. Indeed, a 300 nm thick sputter deposited TiO_2_ film composed of dense nanocrack networks exhibited 3.2 times higher photocatalytic performance in comparison to that of a layer (with identical thickness) prepared by colloidal TiO_2_ NPs. Here, one can see that the morphology achieved by nanocrack networks is significantly different to those achieved in usual sputter deposited TiO_2_ thin films as shown in Figure 6a,b.

### 3.3. Enhancing Nanocrack Formation by Deliberate Choice of Reactive Atmosphere

**Figure 7:**
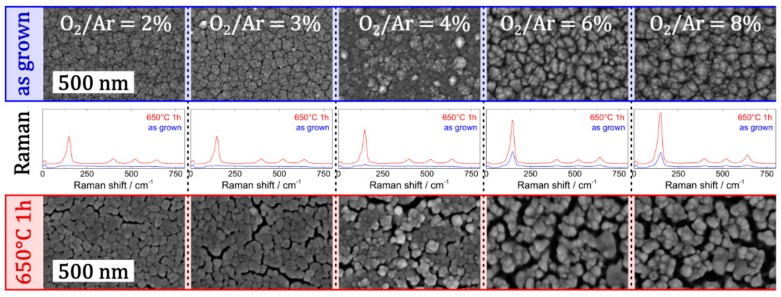
Impact of a variation of O_2_/Ar flow ratio on the morphology and crystallinity of a TiO_2_ thin film deposited by sputter deposition: Top view SEM micrographs (having same scale bar of 500 nm) of the as-grown thin film (upper row) and the thin film with nanocrack networks after heat treatment at 650 °C for 1 h (bottom row). The corresponding Raman spectra are depicted in the middle row. Adapted with permission from [24]. Copyright (2019) IOP.

Complementary to the aforementioned influence of the layer thickness on the extent of nanocrack network formation, the deliberate selection of the reactive atmosphere was also reported as an additional pathway to tailor the morphology of TiO_2_ thin films [24]. As depicted in Figure 7, upon variation of the O_2_/Ar ratio in the reactive gas atmosphere during sputter deposition, the morphology of TiO_2_ thin films can be significantly altered, even at similar layer thicknesses (all thin films exhibit a layer thickness around 500 nm).

The SEM micrographs of the TiO_2_ thin films in the as-grown and heat-treated state indicate a clear trend; a higher oxygen ratio in the reactive atmosphere is reported to result in a lower deposition rate, easier saturation of growing TiO_2_ species on the growth front and consequently more pronounced columnar growth. Interestingly, the choice of the reactive atmosphere also influences the crystallinity of the as-grown thin films, which is indicated by Raman spectroscopy as depicted in Figure 7. While no crystalline features were observed in Raman spectrum of the as-deposited film (at low O_2_/Ar ratio), peaks corresponding to anatase phase started appearing at higher O_2_/Ar ratios. All in all, in the heat-treated state higher oxygen content triggers the formation of a denser network of cracks.

### 3.4. Combining Tailored Sputter Deposited Thin Films and NPs Decoration in a Batch Compatible Process

Following the optimization of the surface area by decorating the top surface with TiO_2_ NPs as described in Section 3, TiO_2_ thin films with dense nanocrack networks can be used as base layers for the fabrication of nanocomposites. In this section, such nanocomposite relying on a nanocrack TiO_2_ thin film and a porous TiO_2_ NPs top layer are discussed. In contrast to the TiO_2_ NPs applied in the context of Section 3.1, here a common chemical solution-based (sol-gel) synthesis approach, which was followed by a standard spin coating process, was used. This approach offers the ability to coat large-area substrates, which is especially attractive with respect to large scale production.

**Figure 8:**
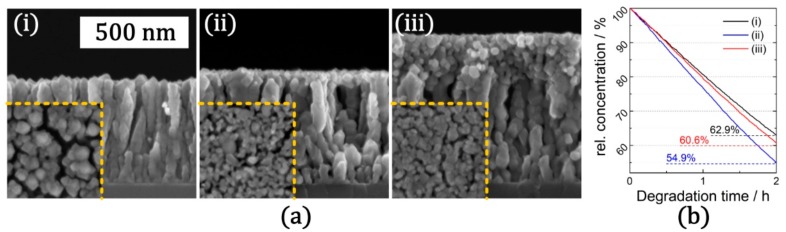
Surface decoration of TiO_2_ thin film (with nanocracks) by TiO_2_ NPs fabricated by sol-gel synthesis: (**a**) SEM micrographs (having same scale bar of 500 nm) in cross section and top view (inset) configuration. (i) Bare TiO_2_ thin film with nanocrack networks after heat treatment; (ii) TiO_2_ thin film with thin NPs layer; (iii) TiO_2_ thin film with thick (180 nm) NPs layer. The corresponding curves for photocatalytic degradation of methylene blue (MB) are depicted in (**b**). (Details are given in Supplementary Materials).

In Figure 8a, cross-sectional SEM micrographs of TiO_2_ bottom layers with different thicknesses ((i) thin and (ii) thick) as well as top TiO_2_ NPs layer (iii) are depicted. The morphology of the undecorated TiO_2_ thin film with the connected network of nanoscopic cracks resembles the morphology of the TiO_2_ thin films described in the previous sections. Upon application of a sol-gel TiO_2_ coating and a consecutive heat treatment step, a film of nanoscopic TiO_2_ particles is attached on top of TiO_2_ columns which forms the base layer. By varying the thickness of the sol-gel coating, the surface decoration either results in a sub-monolayer of TiO_2_ NPs (ii) or a continuous TiO_2_ NPs film (iii). In both cases, after a consecutive heat treatment step, the TiO_2_ NPs top layer and the TiO_2_ base layer were anatase phase and no additional TiO_2_ polymorph was observed.

As depicted in Figure 8b, the performance of the TiO_2_ base layer with nanocrack networks can be further enhanced upon by adding a TiO_2_ NPs top layer. While base layer was able degrade only 37.1% of aqueous MB test solution, nanocomposites with a thin and a thick sol-gel TiO_2_ top layer degraded 39.4% and 45.1%, respectively. Accordingly, especially the nanocomposite with the thin top layer exhibited an extremely high photocatalytic performance. Upon the addition of a sub-monolayer of TiO_2_ NPs, the effective surface area was increased and the underlying structure of the thin film (with nanocracks) stayed accessible to the MB test solution. In the case of the thick top layer, this synergetic effect diminishes as the thickness of the top layer impedes transport of MB to the underlying base layer. As a result, the photocatalytic performance of the nanocomposite with the thick top layer does not increase further and is comparable to the undecorated base layer, rather than to the nanocomposite with the thin top layer.

In conclusion, tailoring the morphology of sputter deposited TiO_2_ thin films by means of nanocrack networks and NPs decoration turns out to be an efficient and simple approach to enhance the photocatalytic performance. In the context of nanocomposites relying on mechanically stable TiO_2_ thin films, the photocatalytic performance could be increased considerably by the addition of TiO_2_ NPs for additional effective surface area. Although such nanocomposites combining different nanostructures of TiO_2_ offer reasonable performance, for further enhancements additional routes apart from the increase in the effective surface area could be explored. These strategies include, amongst others, decorating TiO_2_ thin film with metallic nanostructures which lead to plasmonic and as well as non-plasmonic contributions to the photocatalytic activity. In the following sections, we will focus on the discussion of the latter approach.

## 4. Plasmonic and Non-Plasmonic Enhancement of Photocatalytic Performance by Metallic NPs

**Figure 9:**
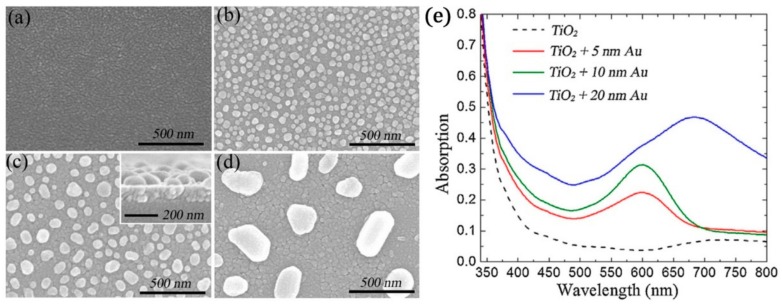
SEM images of (**a**) as-grown 10-nm-thick Au film sputtered on 100-nm-thick TiO_2_ layer and 400 °C annealed composite structures with different initial Au film thickness of (**b**) 5 nm, (**c**) 10 nm, (**d**) 20 nm and (**e**) UV–vis absorption spectra for the prepared TiO_2_ films without and with Au nanoparticles on the surface of TiO_2_. The inset in (c) shows the cross-section SEM image of the Au NPs–TiO_2_ composite structure. Adapted with permission from [43]. Copyright (2014) Elsevier.

Metallic NPs of the so-called free electron metals such as noble metals and aluminum (Al) have extremely large absorption/scattering cross-sections attributed to their ability to generate intense electromagnetic (EM) fields in the nanoscale vicinity of their surface, known as localized surface plasmon resonances (LSPR) [42]. Such plasmonic NPs can be used to broaden and enhance the light absorption of TiO_2_ through scattering, absorption enhancement, sensitization, and hot-electron injection. Chen et al. have shown that decorating sputtered deposited TiO_2_ thin films (as shown in Figure 9) with Au NPs led to a significant enhancement of the photocatalysis [43].

Due to their widespread optical properties, moderate cost (in comparison to other noble metals), and high catalytic efficiency, silver (Ag) NPs are commonly preferred for decorating TiO_2_ to enhance its photocatalytic performance [44]. Besides PVD methods, various wet-chemical synthesis approaches including sol-gel, spray-pyrolysis, chemical reduction, electrochemical deposition, and photo-reduction have been proposed to decorate TiO_2_ with Ag NPs [45]. The photocatalytic nature of TiO_2_ itself has also been reported as an effective tool to deposit Ag NPs under UV irradiation. Although many publications have already presented the photocatalytic deposition of Ag nanostructures on TiO_2_ colloidal particles under UV irradiation, the control of the size and the geometry of Ag particles and their homogenous distribution on such colloidal TiO_2_ particles is still challenging [46].

**Figure 10:**
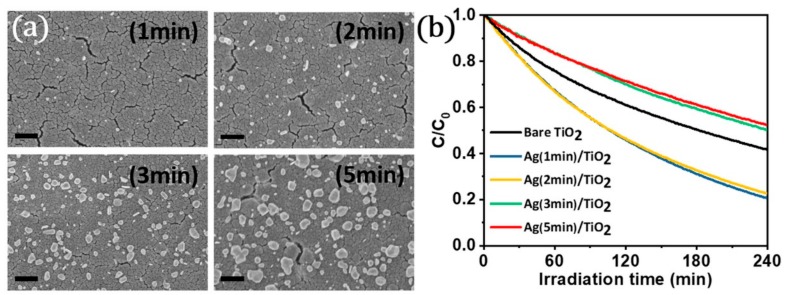
(**a**) SEM images (having same scale bar of 400 nm) of Ag NPs decorated TiO_2_ thin film prepared at different UV exposure time intervals and (**b**) comparison of time-dependent photocatalytic performance for bare and Ag NPs decorated TiO_2_ (with different surface coverages). Adapted with permission from [23]. Copyright (2019) Wiley-VCH.

Recently we reported a facile method which allows highly controllable deposition of Ag NPs, in terms of particle size, shape, and distribution, on TiO_2_ thin film [23]. Different than common approaches, the use of TiO_2_ thin film rather than colloidal TiO_2_ particles allowed the use of a very low-intensity UV light source and this eliminated the risk of particle agglomeration (due to the direct reduction of Ag ions within the solvent) followed by the non-homogenous surface coverage of TiO_2_ by Ag particles. Within a colloid system, TiO_2_ NPs randomly and continuously move, and this hinders the effective exposure to UV light; but an immobilized TiO_2_ surface (thin film) can be activated at a much lower intensity of UV light. One can control the surface coverage of TiO_2_ with Ag NPs by simply altering the deposition time (Figure 10a). Ag NPs with a surface coverage of 1%–4% significantly enhanced the photocatalytic activity of TiO_2_ thin film as shown in Figure 10b. It is believed that electron trapping Ag NPs suppressed the recombination of electron-hole pairs. Here, the enhancement in photocatalytic performance of TiO_2_ by Ag NPs should be considered as a non-plasmonic contribution [47]. The deposition of higher amount Ag NPs on TiO_2_ thin film surface resulted in a decrease in the photocatalytic activity (even lower than the activity of bare TiO_2_ as shown in Figure 10b), which might arise due to the blocking UV light incoming to the TiO_2_ thin film surface. Here, one should keep in mind that Ag is slowly oxidized over time. Alternatively, using Au- and Pt-based metallic NPs may lead to more stable systems to consider [20].

**Figure 11:**
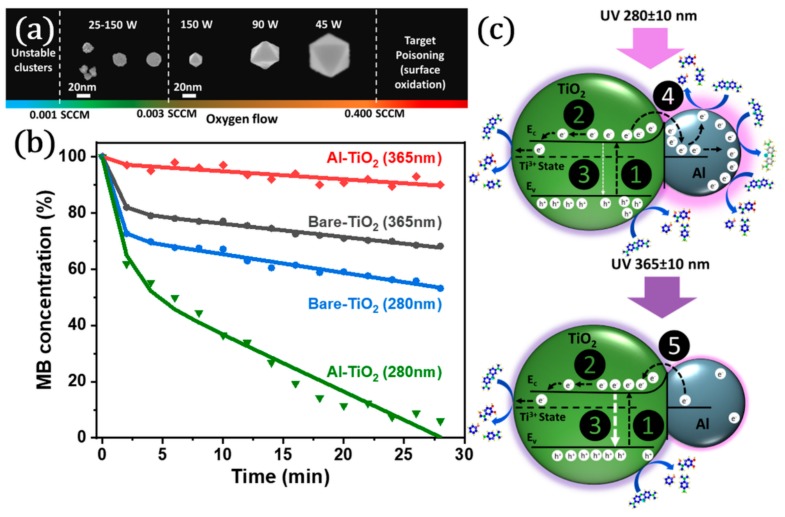
(**a**) Schematic representation of the gas aggregation source (GAS) used to produce Al NPs. (**b**) Photocatalytic degradation of MB solution at 365 ± 10 nm and 280 ± 10 nm UV irradiation by Al-TiO_2_ hybrid structures and (**c**) main mechanisms observed in photocatalytic degradation of MB by Al NPs decorated TiO_2_ thin film at 280 ± 10 and 365 ± 10 nm, where colored purple around Al NPs represent the localized surface plasmon resonance (LSPR). (1) Electron-hole generation. (2) Reduction of Ti^4+^ cations to the Ti^3+^ state. (3) Recombination. (4) Trapping of electrons by Al NPs. (5) Injection of electrons by Al NPs. ((4) and (5) coexist, but depending on the corresponding wavelength, one of them dominates.) Adapted with permission from [31]. Copyright (2018) American Chemical Society.

Recently, Al has been suggested as an alternative plasmonic material in photocatalysis thanks to its extraordinary optical properties. There are only a few studies on the use of Al NPs for UV plasmonic enhancement of TiO_2_ photocatalytic performance since it is challenging to the control the size and the oxidation of reactive Al NPs in comparison to Au and Ag NPs [16]. We presented solvent free deposition of Al NPs on a photocatalytically active sputter-deposited TiO_2_ thin film [31]. We used a GAS to decorate TiO_2_ thin films with Al NPs. During the sputtering process, we added an extremely low amount of oxygen (O_2_) to the main working gas (Ar) to promote the nucleation of Al clusters by binding O_2_ to sputtered Al atoms from the metallic target. By precisely controlling O_2_ flow rate one can easily vary the size and surface coverage of Al NPs on the TiO_2_ thin film (as shown in Figure 11a).

We compared the photocatalytic performance of TiO_2_ before and after decorating it with Al NPs by monitoring the degradation of MB layer deposited on the top surface (rather than immersing the substrate within an aqueous MB solution, which is the most common approach so far). A similar approach (proposing a solid pollutant layer) was given by Chin et al. for monitoring the photocatalytic performance of TiO_2_ coated glass slides [48]. Using such a pollutant layer instead of an aqueous test solution seem to be more surface sensitive, which is critical for analyzing thin films. Actually, alternatively various materials such as oleic acid and fatty molecules (which provide a higher sensitivity in analysis) have been used to monitor the photocatalytic activity [49]. In our analysis, surprisingly, we observed that TiO_2_ layers decorated with 20 nm Al NPs exhibited a lower photocatalytic activity at 365 nm UV irradiation in comparison to the bare TiO_2_ layer (Figure 11b). On the other hand, when we exposed the same surface to a shorter UV wavelength light (280 nm) we observed an extremely high photocatalytic performance (Figure 11b). Since bare TiO_2_ exhibited a clear photocatalytic activity at 280 and 365 nm, electron-hole pairs seemed to be generated in both wavelengths (mechanism 1, Figure 11c). Some photo-generated electrons from TiO_2_ may overcome the Schottky barrier (formed between Al NP and TiO_2_ layer) and be trapped in adjacent Al NP (mechanism 4, Figure 11c). These localized electrons may enhance the degradation of MB by promoting the interaction with surrounding acceptors (Figure 11c). Alternatively, some of the trapped electrons can be injected back to TiO_2_ (mechanism 5, Figure 11c), thus negatively influencing the photocatalytic effect due to the increased electron-hole recombination. At 280 nm the electrons created in TiO_2_ have higher energy, therefore, they can be transferred to Al NPs easily. This seems to enhance the electron-hole separation (less chance for the recombination). In addition, irradiation at 280 nm (matching plasmon frequency) seems to induce hot electrons in the Al NPs which are also involved in photocatalytic reactions. On the other hand, at 365 nm the electrons created in TiO_2_ cannot overcome the Schottky barrier. Here, Al mainly has a surface blocking effect. Even some of the electrons may be transferred to the TiO_2_ enhancing recombination at the Al-TiO_2_ interface. At 365 nm the injection of electrons back to TiO_2_ (mechanism 5) possibly governs and reduces the overall photocatalytic performance. By considering their size and the wavelength of the UV light source, Al NPs can be used effectively for designing cheaper and highly efficient photocatalysts.

## 5. Micro- and Nanostructuring Using Sputter Deposited TiO_2_ Thin Film as a Functional Substrate

**Figure 12:**
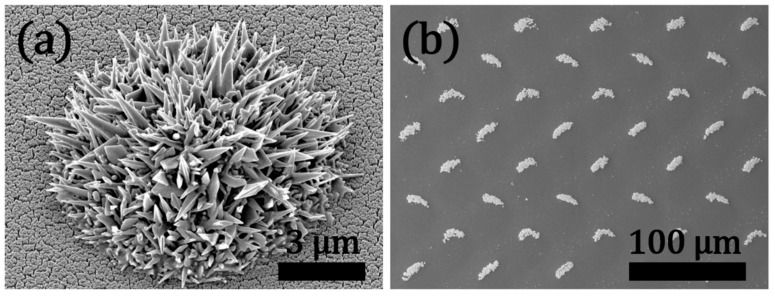
(**a**) Helium ion microscopy (HIM) image of hierarchical Au needle clusters (HAuNC) deposited on a highly activated TiO_2_ thin film and (**b**) SEM image of TiO_2_ thin film patterned with HAuNCs using a simple non-contact polymer mask. Adapted with permission from [53]. Copyright (2018) Wiley-VCH.

The photocatalytic performance of TiO_2_ can be effectively used for the reduction of various metals including Au, Ag, and Cu [50]. Although various research papers are available on the photocatalytic reduction of Au^3+^ ions on colloidal TiO_2_ micro- and nanoparticles (dispersed within a solvent) such an approach is usually far away from being a well-controlled deposition method (in terms of size and geometry control) and is not applicable to positioning or local (selective) loading of Au nanostructures [51]. Recently, we presented a noble method for synthesis of hierarchical Au needle clusters (HAuNCs) on a highly active TiO_2_ thin film by applying a photocatalytic reduction of Au^3+^ without using any capping agent or surfactant [52]. This unconventional approach enables controlling the size and the geometry of deposited HAuNCs by simply altering the photocatalytic activity of the TiO_2_ target (sputter deposited), UV light intensity, and irradiation time. Figure 12a shows a helium ion microscopy (HIM) image of a HAuNC.

The proposed method can be applied for local patterning of TiO_2_ thin films by simply using a non-contact polymer mask as shown in Figure 12b. This may lead to a cost-effective non-lithographic patterning route which can be adapted to various technologies including electrochemical sensor systems and biomedical devices.

## 6. Concluding Remarks

Thanks to their extremely high surface area, colloidal TiO_2_ NPs exhibit high photocatalytic activity, nevertheless, their direct use in environmental remediation such as water treatment is critical since their recovery from such aqueous medium needs advanced and costly technologies. Therefore, it is clear that there is a strong need for highly photocatalytic and as well as robust TiO_2_ thin films for air and water treatment (indoor and outdoor) and similar environmental remediation applications. TiO_2_ colloidal NPs outperform TiO_2_ thin films in terms of the surface area, but by tailoring the surface morphology, one can enhance the photocatalytic performance of TiO_2_ thin films. In this connection, the choice of the deposition method (e.g., reactive sputtering, evaporation, etc.) is a critical issue for the final morphology and as well as the crystallization pathway (for instance getting anatase phase). Decorating sputter deposited TiO_2_ thin films with a top layer of porous TiO_2_ NPs leads to a significant enhancement in the photocatalytic efficiency. One can also directly achieve nanostructured TiO_2_ thin films which possess a high surface area. Combining reactive sputtering at high oxygen partial pressure with subsequent annealing and air-quenching can be a simple and effective method to induce nanocrack networks in sputter deposited TiO_2_ thin films, which help to gain enormous surface area. In addition to the morphological modification, metallic NPs can be effectively used to decorate TiO_2_ thin films to achieve plasmon (LSPR) coupled photocatalysis. In addition to the positive effect of LSPR (enhancement of the photocatalytic performance by hot electrons created on the surfaces of metallic NPs), depending on the irradiation wavelength the LSPR injected hot electrons (from the metallic NPs to TiO_2_) may counteract those transferred from the TiO_2_ to the metallic NPs and this may enhance the recombination in TiO_2_. Beside such plasmonic contributions, metallic NPs can act as an electron-sink which also improves the electron-hole lifetime of TiO_2_. Therefore, metallic NPs can be used to induce both plasmonic and non-plasmonic enhancement of photocatalytic performance. Beside using it in the remediation applications, a highly active TiO_2_ thin film can be used for micro- and nanostructuring processes by simply promoting the photocatalytic reduction of noble metal ions.

## Supplementary Materials

The following are available online at https://www.mdpi.com/1996-1944/12/17/2840/s1.
