# Supplementary material for: Pathways to Tailor Photocatalytic Performance of TiO2 Thin Films Deposited by Reactive Magnetron Sputtering
Source: Materials (Basel). 2019 Sep 3;12(17):2840. doi: 10.3390/ma12172840 (PMC6748074; doi:10.3390/ma12172840)
Supplement: Supplementary file 1 [file materials-12-02840-s001.pdf]

materials

Review

Pathways to Tailor Photocatalytic Performance of TiO_2 Thin Films Deposited by Reactive Magnetron Sputtering

Alexander Vahl, Salih Veziroglu, Bodo Henkel, Thomas Strunskus, Oleksandr Polonskyi, Oral Cenk Aktas * and Franz Faupel *

Institute for Materials Science—Chair for Multicomponent Materials, Faculty of Engineering, Kiel University, Kaiserstraße 2, D-24143 Kiel, Germany
* Correspondence: oca@tf.uni-kiel.de (O.C.A.); ff@tf.uni-kiel.de (F.F.); Tel.: +49-431-880-6225 (F.F.)

Received: 28 July 2019; Accepted: 2 September 2019; Published: 3 September 2019

1. Materials and Methods

1.1. Thin Film Deposition

For the deposition of the TiO_2 thin films, either electron beam evaporation and pulsed unipolar DC magnetron sputtering from a metallic target in a reactive O_2/Ar atmosphere was applied. The details on the deposition of TiO_2 thin films by evaporation are discussed in our previous work [1] and details on deposition by reactive sputtering from a metallic Ti target are described in our earlier publications [1–3].

1.2. Nanoparticle Deposition

The deposition of nanoparticles was realized by following either a sol-gel approach (in the case of TiO_2, as reported by Suresh et al. [4]) or a gas phase synthesis approach (Al and TiO_2) using unipolar DC magnetron sputtering and an in-house gas aggregation source (GAS). Details on the deposition of Al or TiO_2 nanoparticles from GAS can be found in the work of Ghori et al. and Polonskyi et al. respectively [5,6]. For the deposition of TiO_2 nanoparticles from solution a sol-gel approach, 7.18 mL of titanium (IV) butoxide (5593-70-4, purity > 97.0%,Fluka Chemie AG, Buchs, Switzerland) was given into a glass beaker and 12.0 mL acetic acid (64-19-7, purity > 99.8%, Merck KGaA, Darmstadt, Germany) was added as a solvent and protecting agent for the titania precursor under continuous stirring. After 30 min, 37.5 mL deionized water was added drop wise and under vigorous stirring. The solution was subsequently heated on a hot plate (70 °C for 90 min). Afterwards, the solution was transferred into a screw cap flask and stored under continuous stirring until it was coated onto the sputter-deposited TiO_2 thin film by spin coater (WS-650MZ-23NPPB, Laurell Technologies Corporation, North Wales, PA, USA). Consecutively, a heat treatment step (700 °C, 1 h, LE 4/11/R6, Nabertherm GmbH, Lilienthal, Germany) was performed.

1.3. Characterization

In order to achieve a thorough characterization of the fabricated TiO_2 thin films and nanocomposites, atomic force microscopy (AFM, NanoWizard 3, JPK, Berlin, Germany) was applied to obtain information regarding the surface topography and Raman spectroscopy, as well as X-ray diffraction (XRD, Rigaku, Billerica, MA, USA), were applied to study the presence of crystalline TiO_2 polymorphs. In addition, the morphology of the TiO_2 samples was investigated by scanning electron microscopy (SEM, Zeiss Supra 55VP, New York, NY, USA) in top view and cross-sectional configuration and the photocatalytic, UV induced, degradation of organic dyes (at the example of methylene blue, MB) was studied in order to evaluate the photocatalytic performance. Details on the applied thin film characterization methods can be found in our earlier work [1–3].

References

1. Henkel, B.; Neubert, T.; Zabel, S.; Lamprecht, C.; Selhuber-Unkel, C.; Rätzke, K.; Strunskus, T.; Vergöhl, M.; Faupel, F. Photocatalytic properties of titania thin films prepared by sputtering versus evaporation and aging of induced oxygen vacancy defects. *Appl. Catal. B Environ.* **2016**, *180*, 362–371.
2. Henkel, B.; Vahl, A.; Aktas, O.C.; Strunskus, T.; Faupel, F. Self-organized nanocrack networks: A pathway to enlarge catalytic surface area in sputtered ceramic thin films, showcased for photocatalytic TiO_2. *Nanotechnology* **2018**, *29*, 035703.
3. Vahl, A.; Dittmann, J.; Jetter, J.; Veziroglu, S.; Shree, S.; Ababii, N.; Lupan, O.; Aktas, O.C.; Strunskus, T.; Quandt, E.; et al. The impact of O_2/Ar ratio on morphology and functional properties in reactive sputtering of metal oxide thin films. *Nanotechnology* **2019**, *30*, 235603.
4. Suresh, C.; Biju, V.; Mukundan, P.; Warrier, K. Anatase to rutile transformation in sol-gel titania by modification of precursor. *Polyhedron* **1998**, *17*, 3131–3135.
5. Ghori, M.Z.; Veziroglu, S.; Hinz, A.; Shurtleff, B.B.; Polonskyi, O.; Strunskus, T.; Adam, J.; Faupel, F.; Aktas, O.C. Role of UV Plasmonics in the Photocatalytic Performance of TiO_2 Decorated with Aluminum Nanoparticles. *Acs Appl. Nano Mater.* **2018**, *1*, 3760–3764.
6. Polonskyi, O.; Peter, T.; Mohammad Ahadi, A.; Hinz, A.; Strunskus, T.; Zaporojtchenko, V.; Biederman, H.; Faupel, F. Huge increase in gas phase nanoparticle generation by pulsed direct current sputtering in a reactive gas admixture. *Appl. Phys. Lett.* **2013**, *103*.

© 2019 by the authors. Submitted for possible open access publication under the terms and conditions of the Creative Commons Attribution (CC BY) license (http://creativecommons.org/licenses/by/4.0/).
